# Inpatient Palliative Care Is Less Utilized in Rare, Fatal Extrahepatic Cholangiocarcinoma: A Ten-Year National Perspective

**DOI:** 10.3390/ijerph181910004

**Published:** 2021-09-23

**Authors:** Zahra Mojtahedi, Ji Won Yoo, Karen Callahan, Neeraj Bhandari, Donghui Lou, Katayoon Ghodsi, Jay J. Shen

**Affiliations:** 1 Department of Healthcare Administration and Policy, School of Public Health, University of Nevada, Las Vegas, NV 89154, USA; Zahra.mojtahedi@unlv.edu (Z.M.); neeraj.bhandari@unlv.edu (N.B.); ghodsi@unlv.nevada.edu (K.G.); 2 Department of Internal Medicine, University of Nevada Las Vegas School of Medicine, Las Vegas, NV 89154, USA; ji.yoo@unlv.edu; 3 Department of Epidemiology and Biostatistics, School of Public Health, University of Nevada, Las Vegas, NV 89154, USA; karen.callahan@unlv.edu; 4 The First Hospital of Qinhuangdao, Qinhuangdao 066001, China; loudonghui72@163.com

**Keywords:** extrahepatic cholangiocarcinoma, palliative care, LOS, hospital charges, public health

## Abstract

Background—Extrahepatic cholangiocarcinoma (ECC) is a rare, morbid, fatal cancer with distressing symptoms. Maintaining a high quality of life while reducing hospital charges and length of stay (LOS) for the end-of-life period remains a major challenge for the healthcare system. Palliative care utilization has been shown to address these challenges; moreover, its use has increased in recent years among cancer patients. However, the utilization of palliative care in rare cancers, such as ECC, has not yet been explored. Objectives—To investigate palliative care utilization among ECC patients admitted to US hospitals between 2007 and 2016 and its association with patient demographics, clinical characteristics, hospital charges, and LOS. Methods—De-identified patient data of each hospitalization were retrieved from the National Inpatient Sample (NIS) database. Codes V66.7 (ICD-9-CM) or Z51.5 (ICD-10-CM) were used to find palliative care utilization. Multivariate adjusted logistic regression analyses were conducted to assess factors associated with palliative care use, LOS, hospital charges, and in-hospital death. Results—Of 4426 hospitalizations, only 6.7% received palliative care services. Palliative care utilization did not significantly increase over time (*p* = 0.06); it reduced hospital charges by USD 25,937 (*p* < 0.0001) and LOS by 1.3 days (*p* = 0.0004) per hospitalization. Palliative care was positively associated with female gender, severe disease, and age group ≥80 (*p* ≤ 0.05). The average LOS was 8.5 days for each admission. Conclusions—Hospital admissions with palliative care utilization had lower hospital charges and LOS in ECC. However, ECC patients received less palliative care compared with more common cancers sharing similar symptoms (e.g., pancreatic cancer). ECC patients also had longer LOS compared with the national average. Further research is warranted to develop interventions to increase palliative care utilization among ECC hospital patients.

## 1. Introduction

Cancer patients and their families, especially those coping with end-stage disease, often simultaneously face a poor quality of life and a considerable financial burden from hospital charges. Moreover, some intensive interventions for end-stage cancer patients do not produce better outcomes and merely exacerbate the financial burden [1,2]. These charges, as well as significant discomfort, could potentially be avoided by engagement with palliative care [1,2], whereby an expert interdisciplinary team consisting of physicians, nurses, social workers, and other specialties work together to alleviate pain as well as psychological and spiritual distress of patients struggling with terminal diseases [3,4]. Palliative care has been associated with reducing hospital charges and length of hospital stay (LOS) [1,5,6] since the management of symptoms avoids unnecessary charges and long hospital stays [1,2,3,4].

Palliative care utilization has significantly increased in recent years, especially for common poor-prognosis cancers such as pancreatic and lung cancers; however, usage varies by gender, race/ethnicity, educational level, obesity, socioeconomic status, and health insurance type [5,6,7,8,9]. Additionally, cancer patients whose physicians have discussed end-of-life planning are more likely to choose palliation over aggressive care [1,2]. Palliative care usage also varies widely based on tumor type [10], with documented utilization (2005 to 2014) ranging from 4.9% in good prognosis cancers such as breast cancer, to 16% in liver cancer [10]. Reasons have not been explored thoroughly, but the variation may be related to differences in prognosis of the tumor, distressful symptoms, or patient demographics. Regardless, palliative care usage for rare, usually fatal, cancers such as extrahepatic cholangiocarcinoma (extrahepatic bile duct cancer; ECC) has not yet been studied.

In the US, the approximate annual incidence of ECC is 0.44 cases per 100,000 [11]. The disease is highly aggressive, usually presenting at an advanced stage [12,13] when biliary tract obstruction has caused overt jaundice and distressful symptoms, similar to the more common pancreatic cancer [14,15]. Because of tumor inoperability and the poor prognosis, ECC patients could be ideal candidates for palliative care services that would relieve pain and improve their quality of life.

Palliative care can be initiated at any time during terminal illnesses alongside curative therapies. It is called hospice care if palliation is provided to the patients with terminal diagnoses who no longer have the option or do not choose the option of active treatment in the last six months of life, often provided at home, but it can be in any setting [1,2,3,4]. Hospitals are the most common setting for non-hospice palliative care services [16]; information from a representative sample of all US hospital stays is available in the National Inpatient Sample dataset (NIS). Diagnoses and procedures during each hospital stay are recorded in the NIS dataset using the International Classification of Diseases (ICD) codes [17]. Patients, at discharge, are coded for palliative care when terms such as palliative care, comfort care, end-of-life care, hospice, or similar terms are written in the record [18]. The NIS data also facilitate reliable comparisons across studies. Using the NIS data, previous research found that 12% of pancreatic cancer and 16% of liver cancer hospitalizations from 2005 to 2014 utilized inpatient palliative care [10]. As ECC and pancreatic and liver cancer patients are somehow similar demographically and experience similarly distressing symptoms [19], a reasonable estimation during the same period for palliative care utilization among ECC patients would approximate above 10% as well; however, to the best of our knowledge, this has not yet been studied.

In the current study, using V66.7 (ICD-9, before October 2015) and Z51.5 (ICD-10, after October 2015) codes within the NIS database, we aimed to characterize the extent of utilization of inpatient palliative care services among ECC patients and the influence of several factors on this usage, including gender, age, race/ethnicity, in-hospital death, LOS, hospital charges, payer source, and severity of the disease. We further examined the influence of palliative care usage on hospital charges, LOS, and in-hospital death.

## 2. Methods

### 2.1. Data Source and Study Design

A retrospective, cross-sectional study was performed using discharge data from the NIS database, developed for the Healthcare Charge and Utilization Project (HCUP). The NIS database, encompassing data from over 7 million hospital stays annually, represents a 20% sample of all hospitals in 44 US states. Upon completion of a data user agreement with the Agency for Healthcare Research and Quality (sponsoring agency for HCUP), completely de-identified data were delivered for each hospital stay. The Institutional Review Board at the University of Nevada Las Vegas reviewed the study proposal and deemed it Exempt.

### 2.2. Study Population and Variables

Included patients were aged 18 years or older, in the hospital between 2007 and 2016, with ECC as the principal diagnosis, identified using the International Classification of Diseases, and Clinical Modification codes of 156.1 for ICD-9CM and C24 for ICD-10-CM. Extracted demographic variables of interest for each patient included age, gender, race, and insurance payer type (Medicare, Medicaid, private insurance, other); clinical variable included the severity of illness, based on the All Patient Refined Diagnosis-Related Group (APRDRG); scoring hospital variables included LOS, in-hospital death, and total hospital charges. The severity of illness has 4 levels of 1 to 4, indicating minor, moderate, major, and extreme loss of function. Receipt of palliative care services in hospitals was indicated by codes V66.7 for ICD-9-CM or Z51.5 for ICD-10-CM. Total hospital charges were adjusted for annual health growth [20,21].

### 2.3. Statistical Analyses

Generalized regression analyses adjusted for demographic, clinical, and hospital characteristics were conducted for four main outcomes: receipt of palliative care, LOS, hospital charges, and death while in the hospital. Palliative care receipt was also an independent variable in predicting LOS and hospital charges. Odds ratios (ORs) and their corresponding 95% confidence intervals (CIs) were computed. All analyses were performed using SAS statistical software version 9.4 (SAS Institute Inc, Cary, NC, USA). All reported *p*-values were 2-tailed; *p*-values < 0.05 were considered statistically significant.

## 3. Results

The descriptive analysis of the current study has been demonstrated in Table 1. A total of 4426 ECC hospitalizations between 2007 and 2016 were included in the study; the weighted number of cases was estimated to be 22,028. Inpatient palliative care utilization was recorded in 6.7% of ECC hospitalizations during the study period. This percentage climbed to 32.9% in a subset of hospitalizations that ended in death (5.4% of all patients). The mean age of patients was 70.7 ± 27.6; 56.2% were 70 or older. Most were men (55.2%), White (70.8%), had Medicare (64.3%), and major or extreme illness severity (66.8%) The mean length of stay was 8.5 days; mean total hospital charges were USD 83,042 (Table 1). Palliative care utilization for ECC did not significantly increase over the study period (OR = 1.19, CI = 0.99–1.42; *p* = 0.06). Factors associated with a higher receipt of palliative care included age older than 80 years compared with patients aged 50–60 years old (OR = 2.13, *p* = 0.018), disease severity (OR = 2.39 as severity level increased by one, *p* < 0.001), being female, (OR = 1.38, *p* = 0.05), and dying in the hospital (OR = 8.46, *p* < 0.001). Neither race/ethnicity nor payer source were associated with palliative care utilization (Table 2).

Among ECC patients, the average LOS decreased by 0.14 days over time (*p* = 0.0004). Palliative care utilization significantly reduced LOS by 1.3 days. Factors associated with longer LOS included being Black, with 1.17 day’s increase compared with White patients, (*p* = 0.0017), the severity of illness (increases of 3.84 days for each increase in severity level, *p* < 0.0001), and being insured with Medicaid, with increases of 2.55 days compared with those who were privately insured, (*p* < 0.0001), although having Medicare reduced LOS compared with the privately insured (0.68 days; *p* = 0.0281) (Table 3).

No significant change in hospital charges over time for ECC patients was observed after adjustment for the health inflation rate (*p* = 0.2826). Palliative care was associated with a reduction of USD 25,937 per hospitalization (*p* <0.0001). Female patients incurred lower charges than male patients (−USD 7486; *p* = 0.0005). Higher hospital charges were associated with Asian/Pacific Islanders and Hispanics compared with White patients (USD 16,148, *p* = 0.0013 and USD 12,045, *p* = 0.0011, respectively). Regarding Black patients, their hospital charges were not significantly different from their White counterparts (USD 1108; *p* = 0.77). Disease severity (USD 31,986 per one level higher; *p* < 0.0001) also increased hospital charges (Table 4).

The proportion of ECC patients who died in the hospital decreased significantly over time (OR = 0.89, *p* = 0.048). In-hospital death was strongly associated with palliative care (OR = 8.94, *p* < 0.0001). In-hospital death was associated with age: those from ages 70–79 had 3.31 times greater odds of death in hospital than those aged 50–59 (*p* = 0.0011); for those ages 80 plus years, odds of death in the hospital were 2.56 times higher (*p* = 0.0120). Disease severity also increased odds of death in hospitals by 4.81 for each level of severity increase (*p* < 0.0001). Medicare beneficiaries had lower odds of death in hospitals compared with patients with private insurance (OR = 0.54, *p* = 0.0214) (Table 5).

## 4. Discussion

In the current study, we present the first examination of trends of palliative care utilization among patients with the rare but deadly ECC. We found that only 6.7% of ECC hospitalizations utilized palliative care services between 2007 and 2016, which was much lower than anticipated, and lower than palliative care use for other similar poor prognosis cancers, such as pancreatic and liver cancer, in the US [10]. However, similar to findings with other diseases [5,6,7,22], we also found that palliative care utilization significantly reduced hospital charges and LOS for the ECC patients. Reasons for the reductions in hospital charges include the effective management of the symptoms, the performance of fewer aggressive procedures, and shorter stays in the hospital for patients who receive palliative care services [23].

Prior studies, using the NIS dataset, documented palliative care utilization by 12% of pancreatic cancer, 16% of liver cancer, and 9.9% of overall cancer hospitalizations in the USA from 2005 to 2014 [10]. Our finding of lower utilization of palliative care in ECC patients compared with overall cancer patients cannot be due to differences in the study periods since palliative care utilization has been increasing over time and our study is more recent [10]. Our study found not only that palliative care was utilized less among ECC patients but also that there were no significant increases over time. Although differences in palliative care utilization by tumor type have been documented in prior studies [10], efforts should be made to increase palliative care utilization among ECC patients to at least the level used in pancreatic cancer, a malignancy most similar in presentation, symptoms, and prognosis.

Consistent with prior studies in US hospitals [5,6,7] and across the world [24], we found that palliative care utilization was significantly higher among women as well as patients older than 80 years and with a more severe disease. The age group of 80 years plus might have also received more outpatient palliative care services since they had lower odds of dying in hospital compared with the age group of 70–79 years. Older age and disease severity could be criteria for recommending palliative care services when curative procedures are no longer options. Interestingly, we also found that female patients had USD 7486 lower hospital charges than men, which may reflect higher usage of palliative care by women, although it could also reflect known gender differences in national inpatient stay charges, with mean costs per stay for men at USD 13,300 compared with only USD 10,500 for women [17]. Among patients with more severe diseases, palliative care utilization was higher but total charges were also higher, which might be due to their longer hospital stay due to more severe disease.

The current study found that palliative care utilization was significantly higher among patients who died in the hospital than those who were discharged. Although palliative care is ideally provided throughout the disease course, research has shown that most patients referred to inpatient hospital palliative care services were at the end of their life [5,6,7]. Thus, greater utilization of palliative care by ECC patients who died in the hospital indicates that inpatient palliative care services were happening extremely proximal to death, a finding consistent with many previous studies of palliative care among cancer patients in hospitals [5,6,7]. Certain policies, educating physicians and patients, are needed to change the timing of the provision of palliative care services since the aim of palliative care is to provide comfort during the end-of-life period, not just to manage death [25]. Schoenherr et al. investigated trends in hospital-based palliative care in the US from 2013 to 2017 [26]. They found that the percentage of patients who received palliative care services and were subsequently discharged increased over the study period, indicating that palliative care teams may have been seeing patients earlier in their illnesses. Moreover, this suggests that palliative care teams were increasingly effective at facilitating a safe transition from the hospital to home or hospice centers, a practice consistent with guidelines recommending palliative care [26] and supported by the Affordable Care Act (ACA) [27]. Home death has been documented in prospective cohort studies to be preferred by the majority of cancer patients as long as their family caregivers did not have a high burden [28]. Thus, health managers and policymakers should develop policies that honor those preferences.

The average LOS for all US hospital stays was 4.6 days in 2016 [17]. Reducing LOS in recent decades has been observed in many health care systems across several countries aiming to improve efficiency [29]. Although in the current study, ECC patients significantly decreased their length of stay in hospitals over time, the average LOS was 8.5 days, longer than national averages possibly due to the nature of this particular poor prognosis cancer and less palliative care utilization. Our finding that LOS was significantly higher among Black ECC patients is consistent with prior national studies, which found higher LOS among Black cancer patients compared with White patients [3]. Moreover, the longer LOS documented in the current study for Medicaid and Medicare beneficiaries seems to be in line with national trends [18]; both are not specific to ECC and should be addressed in the nation.

The current study is the first to evaluate inpatients’ palliative care receipt among ECC patients using the largest publicly available inpatient hospital database. We found that ECC patients utilized palliative care services less compared with patients with similar cancers and the national average [10]. ECC patients also had longer LOS compared with the national average [17]. Underutilization of palliative care [30] and long LOS [31] have both been recognized as a disparity in the healthcare system, implying that ECC patients need more attention and care.

Palliative care can be improved by educating health providers, patients, and the public. Health providers should offer palliative care to all eligible patients regardless of diagnosis and incidence of the disease. Training of palliative care specialists is also crucial for this improvement since there is a shortage of specialists in this area [32]. Public awareness about palliative care can be enhanced through social media, news, and documentary in a way that palliative care becomes public knowledge. Patients should not become aware of such valuable care just at the end of life. Governments should identify policies for the promotion of palliative care, including workforce growth, payment reform, enhancing public awareness, as well as research grants. Since the benefits of palliative care are becoming more obvious in recent years, and with increasing numbers of grants and incentives [33], it is anticipated that palliative care utilization for all eligible patients will be increased in upcoming years—though it still might be suboptimal.

Our study is subject to some limitations. We used ICD-9-CM and ICD-10-CM codes for the identification of palliative care services; these could potentially be inaccurate due to incorrect data entry [34]. The NIS database does not contain tumor stage, grade, or subtype information, which might be an addition to the disease severity. Moreover, our study includes only inpatient hospital data; the NIS does not contain information about palliative care utilization in other settings. The NIS also provides de-identified data on each admission, so readmitted cases are considered as new admissions, and hospital charges and LOS are calculated for each admission not for each patient. ECC may not be fatal in rare cases [35]. Therefore, another limitation of our study is that we did not exclude possible rare curable ECC cases who might not have been eligible for palliative care. The large numbers of our cases could minimize the effects of this limitation on our findings.

## 5. Conclusions

In conclusion, the current study, encompassing ten years of data from the NIS database studying ECC patients of diverse race/ethnicities, is unique as we believe it is the first to study palliative care trends among these patients. Due to the quality of the NIS dataset, we believe that the findings in the current study are generalizable to most ECC patients in US hospitals. Our findings of extremely low palliative care usage for patients suffering from this rare but lethal cancer are troubling, and warrant interventions to increase time provision of palliative care services among ECC patients in US hospitals.

## Figures and Tables

**Table 1:** Demographic, clinical, and hospital characteristics of 22,028 ECC hospitalizations (weighted national estimate) in US hospitals (2007–2016; NIS Data) *.

Characteristics		2007–2016	2007	2010	2013	2016
Age in years (mean ± SD)		70.7 ± 27.6	71.1 ± 29.4	71.0 ± 26.3	70.0 ± 28.4	70.1 ± 29.0
Age group	<50	1157 (5.2)	106 (6.0)	109 (3.9)	155 (6.6)	130 (6.3)
	50–59	2826 (12.9)	275 (15.7)	378 (13.4)	264 (11.3)	250 (12.2)
	60–69	5629 (25.6)	352 (20.1)	746 (26.4)	664 (28.3)	524 (25.6)
	70–79	6374 (29.0)	457 (26.0)	846 (29.9)	639 (27.3)	599 (29.3)
	≥80	5974 (27.2)	564 (32.2)	744 (26.3)	619 (26.4)	544 (26.6)
Gender	Female	9842 (44.8)	781 (44.4)	1258 (44.5)	1039 (44.4)	879 (43)
	Male	12,119 (55.2)	978 (55.6)	1566 (55.6)	1305 (55.6)	1170 (57)
Race/Ethnicity	White	15,551 (70.8)	1291 (73.4)	1964 (69.6)	1714 (73.1)	1445 (70.5)
	Black	1980 (9.0)	166 (9.5)	305 (10.8)	254 (10.9)	160 (7.8)
	Hispanic	2299 (10.4)	197 (11.2)	328 (11.6)	239 (10.2)	239 (11.7)
	API	1114 (5.0)	61 (3.5)	142 (5.0)	45 (1.9)	100 (4.9)
	Other	1015 (4.6)	43 (2.4)	83 (3.0)	89 (3.8)	104 (5.2)
Insurance/Payer Source	Medicare	14,123 (64.3)	1139 (64.8)	1814 (64.2)	1534 (65.5)	1335 (65.1)
	Medicaid	1528 (6.9)	108 (6.2)	167 (5.9.5)	195 (8.3)	170 (8.3)
	Private insurance	5276 (24.0)	414 (23.5)	663 (23.5)	535 (22.8)	460 (22.4)
	Self-pay	547 (2.4)	54 (3.09)	109 (3.9)	34 (1.5)	44 (2.2)
	Other	486 (2.2)	42 (2.43)	69 (2.5)	45 (1.9)	39 (2.0)
Severity of illness (APR-DRG)	0–1, lowest	893 (4.0)	116 (6.6)	162 (5.7)	54 (2.3)	94 (4.6)
	2, moderate	6417 (29.2)	536 (30.4)	838 (29.7)	640 (27.3)	749 (36.6)
	3, major	11,118 (50.7)	836 (47.6)	1352 (47.9)	1349 (57.6)	929 (45.4)
	4, extreme	3532 (16.1)	270 (15.4)	471 (16.7)	299 (12.8)	275 (13.4)
Received palliative care services		1478 (6.7)	63 (2.5)	180 (6.3)	190 (8.1)	184 (9.0)
In-hospital death		1196 (5.4)	139 (7.9)	174 (6.1)	80 (3.4)	84 (4.1)
Of these, received palliative care		394 (32.9)	24 (17.3)	58 (33.6)	40 (50.0)	40 (47.0)
LOS in days (mean ± SD)		8.5 ± 17.4	9.3 ±19.5	8.6 ± 16.6	8.0 ± 16.0	7.6 ± 14.2
Total charges in USD (mean ± SD)		83,042 ± 217,330	64,128 ± 159,902	83,048 ± 194,879	82,765 ± 191,909	96,063 ± 217,468

* *n* (%), unless otherwise indicated. Abbreviations: API, Asian/Pacific Islander; APR-DRG, All Patient Refined Diagnosis-Related Group; CI, confidence interval; SD, standard deviation.

**Table 2:** Factors associated with receipt of inpatient palliative care services in ECC in US hospitals. NIS Data (2007–2016).

		Odds Ratio	95% CI	*p*-Value
Year		1.19	0.99–1.42	0.060
Age group	50–60	reference		
	<50	0.67	0.28–1.62	0.378
	60–70	0.68	0.37–1.24	0.212
	70–80	0.79	0.42–1.50	0.480
	>80	2.13	1.13–4.02	0.018
Gender	Male	reference		
	Female	1.38	1.00–1.90	0.050
Race/Ethnicity	White	reference		
	API	0.73	0.33–1.62	0.450
	Black	0.96	0.55–1.68	0.894
	Hispanic	0.82	0.47–1.45	0.508
Insurance/Payer Source	Private insurance	reference		
	Medicaid	0.84	0.41–1.71	0.632
	Medicare	0.72	0.44–1.18	0.198
	Self-pay	1.22	0.44–3.38	0.696
Severity of illness: APR-DRG		2.39	1.82–3.13	<0.001
Died in hospital		8.46	4.51–15.86	<0.001

Abbreviations: API, Asian/Pacific Islander; APR-DRG, All Patient Refined Diagnosis-Related Group; CI, confidence interval.

**Table 3:** Factors associated with LOS (length of stay) in ECC in US hospitals from 2007 to 2016. NIS Data.

		Coefficient, β	Standard Error	*p*-Value
Year		−0.14	0.03	0.0004
Received palliative care services		−1.3	0.42	0.0023
Age group	50–60	reference		
	<50	0.67	0.54	0.2118
	60–70	0.55	0.37	0.1426
	70–80	0.69	0.42	0.1001
	>80	−0.16	0.43	0.6996
Gender	Male	reference		
	Female	−0.26	0.21	0.2177
Race/Ethnicity	White	reference		
	API	0.75	0.48	0.1234
	Black	1.17	0.37	0.0017
	Hispanic	0.46	0.35	0.1891
Insurance/Payer Source	Private insurance	reference		
	Medicaid	2.55	0.46	<0.0001
	Medicare	0.68	0.31	0.0281
	Self-pay	1.23	0.69	0.0761
Severity of illness: APR-DRG		3.84	0.14	<0.0001

Abbreviations: API, Asian/Pacific Islander; APR-DRG, All Patient Refined Diagnosis-Related Group.

**Table 4:** Factors associated with hospital charges in ECC in US hospitals from 2007 to 2016. NIS Data.

		Coefficient, β	Standard Error	*p*-Value
Year		USD 487	USD 453	0.2826
Received palliative care services		USD 25,937	USD 4378	<0.0001
Age group	50–60	reference		
	<50	USD 5576	USD 5440	0.3055
	60–70	USD 5948	USD 3774	0.1151
	70–80	USD 8186	USD 4207	0.0518
	>80	USD (3047)	USD 4335	0.4822
Gender	Male	reference		
	Female	USD 7486	USD 2132	0.0005
Race/Ethnicity	White	reference		
	API	USD 16,148	USD 4998	0.0013
	Black	USD 1108	USD 3790	0.77
	Hispanic	USD 12,045	USD 3678	0.0011
Insurance/Payer Source	Private insurance	reference		
	Medicaid	USD 7302	USD 4627	0.1147
	Medicare	USD 1076	USD 3147	0.7325
	Self-pay	USD 1262	USD 6999	0.856
Severity of illness: APR-DRG		USD 31,986	USD 1469	<0.0001

Abbreviations: API, Asian/Pacific Islander; APR-DRG, All Patient Refined Diagnosis-Related Group.

**Table 5:** Factors associated with in-hospital death in ECC in US hospitals from 2007 to 2016. NIS Data.

		Odds Ratio	95% CI	*p*-Value
Year		0.89	1.01–1.23	0.0480
Received palliative care services		8.94	5.74–13.94	<0.0001
Age group	50–60	reference		
	<50	0.65	0.20–2.13	0.4856
	60–70	1.64	0.83–3.25	0.1518
	70–80	3.31	1.61–6.82	0.0011
	>80	2.56	1.23–5.36	0.0120
Gender	Male	reference		
	Female	0.94	0.66–1.33	0.7400
Race/Ethnicity	White	reference		
	API	0.86	0.38–1.96	0.7315
	Black	1.11	0.62–1.97	0.7145
	Hispanic	0.95	0.52–1.72	0.8750
Insurance/Payer Source	Private insurance	reference		
	Medicaid	1.16	0.52–2.55	0.7103
	Medicare	0.54	0.32–0.91	0.0214
	Self-pay	1.37	0.49–3.82	0.5469
Severity of illness: APR-DRG		4.81	3.57–6.48	<0.0001

Abbreviations: API, Asian/Pacific Islander; APR-DRG, All Patient Refined Diagnosis-Related Group; CI, confidence interval.

## Data Availability

The data of the current study are available upon request from the corresponding author.
